# P-1027. Evaluating the Characteristics of Catheter-Associated Urinary Tract Infections at University Health Kansas City

**DOI:** 10.1093/ofid/ofaf695.1223

**Published:** 2026-01-11

**Authors:** Chia-Chi A Fu, Eryiel Mascardo, Melia Stolberg, An-Lin Cheng, Ume Abbas

**Affiliations:** University of Missouri - Kansas City, Kansas City, Missouri; University of Missouri - Kansas City, Kansas City, Missouri; University Health Kansas City, Kansas City, Missouri; University of Missouri-Kansas city, Kansas city, Missouri; University of Missouri-Kansas City, Kansas City, Missouri, Kansas City, Missouri

## Abstract

**Results:**

In 2023, there were 17 CAUTI cases compared to 9 in 2024. There were no statistically significant (p< 0.05) differences between the two groups in terms of continuous (mean/median) or categorical variables including: age (65.6 vs 58.5 yrs); BMI (24.6 vs 28), CCI, (4.5 vs 4.7); LOS (27 vs 35 days); hospital day of CAUTI diagnosis (22 vs 14); number of re-catheterizations (3.5 vs 1); and catheter duration, (10 vs 7 days). The most common indication for ordering urinalysis with reflex to culture or urine culture was fever which decreased from 64.7% in 2023 to 42.9% in 2024. More than half of cases in both years were prescribed opioids and/or bowel regimens. In 2023, the median CAUTI rate was 2.05 infections per 1000 catheter days and median SIR was 2.415, which decreased to 1.83 and 1.308, respectively in 2024. However, these decreases were not statistically significant.

**Conclusion:**

There was a trend towards decrease in CAUTI rates and SIR in 2024. However, the features and metrics of CAUTI were not significantly different between the years compared. Fever was the most common indication for urinalysis and urine culture. Additional interventions including diagnostic stewardship would be required for effective and durable CAUTI prevention.

**Disclosures:**

All Authors: No reported disclosures

**Background:**

Catheter associated urinary tract infections (CAUTI) are a substantial burden on healthcare. University Health Kansas City is a 238-bed safety net hospital where CAUTI rates and standardized infection ratios (SIR) have been high for several years. Various interventions were implemented for infection prevention with an apparent decrease in CAUTIs in 2024. We evaluated the features and metrics of CAUTI cases in 2023 compared to 2024.

**Methods:**

In a retrospective cohort study, we extracted data from inpatient encounters identified with CAUTI events in 2023 and 2024. Data included demographic information, Charleson Comorbidity Index (CCI), BMI, length of stay (LOS), timeline of CAUTI diagnosis, number of re-catheterizations, urine culture indication, clinical findings, and medications. Continuous variables were compared using Mann-Whitney U or t tests, while X^2^ or Fisher’s exact tests were used for categorical variables. Analyses of CAUTI rates and SIR were performed using control charts with two timelines; 2023 vs 2024 and 01/23-03/24 vs 04/24-03/25.

